# Editorial: Retinal biomarkers of neurodegenerative diseases

**DOI:** 10.3389/fopht.2024.1479755

**Published:** 2024-10-07

**Authors:** Sieun Lee, Joanne Aiko Matsubara

**Affiliations:** ^1^ Mental Health and Clinical Neurosciences, School of Medicine, University of Nottingham, Nottingham, United Kingdom; ^2^ Department of Ophthalmology & Visual Sciences, Faculty of Medicine, University of British Columbia, Vancouver, BC, Canada

**Keywords:** retina, Alzheimer’s diseae, biomarker, neurodegenerative diseases, OCT, ergothioneine, oculometric indicators, OCTA

The intersection of ophthalmology and neurology has opened a promising avenue in the early detection and monitoring of neurodegenerative diseases. Unlike the brain which is enclosed in the skull, the retina can be directly imaged through the pupil using light-based techniques. With the rapid adoption of optical coherence tomography (OCT) over the last decades, the retina is now routinely imaged in clinics in high-resolution 3D volumes containing rich information not only about the health of the retina itself but also that of the overall nervous and vascular systems including the brain. This advancement has sparked energetic research for identifying retinal signatures of various neurodegenerative diseases, including dementia (Alzheimer’s disease, frontotemporal dementia, Lewy body dementia), movement disorders (Parkinson’s disease, Huntington’s disease), and multiple sclerosis (MS). (1, 2) Among these, MS has well-known retinal inflammatory symptoms such as optic neuritis, uveitis, and vasculitis. Other diseases that have been primarily associated with neurodegeneration in the brain have also been shown to affect the eye, most often retinal thinning observable using OCT. However, the sensitivity, specificity, and timing of these ocular manifestations, as well as the effect of treatments, remain open questions and subject of further research.

Under the Research Topic “*Retinal Biomarkers of Neurodegenerative Diseases*,” cross-listed in Frontiers of Ophthalmology, Frontiers of Neuroscience, and Frontiers of Aging Neuroscience, we have published a small but illuminating Research Topic of original research articles and a systematic review that showcases the current state of this research area that has been garnering much attention in recent years.

As the most prevalent type of dementia, Alzheimer’s Disease (AD) continues to be a focus for retinal biomarker research. AD currently affects 1 in 6 of those aged 80 or above and its impact will likely grow with the ageing global population. Diagnosis of AD is based on cognitive functional deficit, although the molecular and structural signatures precede it by decades. Common scepticism about the value of an early biomarker of AD has been the lack of effective treatment. However, with the recent advances in amyloid beta (Ab)-targeting drugs, early diagnosis is more important than ever, in addition to the improved quality of care and support for the patients and caregivers.

Chen et al. performed a meta-analysis of 9 studies on the association between retinal layer thickness and hippocampal volume in normal control (NC), mild cognitive impairment (MCI), and AD, and found that there was a significant positive correlation between retinal nerve fibre layer thickness and hippocampal volume throughout all groups and that the correlation was much higher in the NC group than the AD group. The result suggests that there are both normative and pathological relationships between the retinal and hippocampal morphometrics, and while encouraging, poses the question of handling inter-individual variability and specificity of retinal layer thickness as a proxy marker of pathological hippocampal thinning. Marquie et al. examined the association between retinal vessel density from OCT angiogram (OCTA) and cerebral spinal fluid (CSF) Ab and phosphorylated tau measure in the NORFACE cohort in Spain. The authors found that the retinal vessel density parameters were not statistically significantly associated with the AT(N) groups of Normal, Alzheimer, and Suspected Alzheimer pathology or the CSF measures. This thorough study of a large cohort highlights among others the importance of publishing null results, the importance of nuance in the type and specification of retinal biomarkers to be clinically useful, and the challenge of considering a peripheral disease marker in comparison to another marker that is more established and considered to be closer to the central pathophysiology.

Wijesinghe et al. conducted a novel, ambitious study that delves into both the disease mechanism and potential treatment of AD by investigating the neuroprotective effect of ergothioneine (Ergo), a naturally occurring dietary antioxidant, on Ab clearance in an AD mouse model (5XFAD). The study found that the Ergo-treated mice had significantly lower Ab immunoreactivity, a lower number of visible Ab deposits, and an increased number of IBA1+ blood-derived phagocytic macrophages. Disrupted Ab clearance is a central process in AD pathophysiology and treatment target, and understanding the role and dysfunction of glial cells in Ab clearance in AD is crucial. The study also is a great example of using the retina in research for developing novel drugs for neurodegenerative diseases.

In a different topic, Berneshawi et al. examined a set of oculomotor measurements (oculometrics) in asymptomatic systemic lupus erythematosus (SLE) patients undergoing long-term hydroxychloroquine treatment which carries a risk of retinal toxicity. Interestingly, the SLE patients without any symptoms showed a significant reduction in multiple oculomotor functions in comparison to age-matched healthy controls. This potentially has important clinical implications as hydroxychloroquine retinopathy is asymptomatic in the early stage, and the resulting cellular damage is irreversible and may continue for some time even after the treatment is ceased, making early detection paramount. Furthermore, several of the oculometrics were linearly correlated with retinal thickness in the patients. Structure-function relationship is a major Research Topic in ophthalmology and visual science, and such a multi-modal/multi-domain approach will contribute to gaining novel insights into disease mechanisms as well as developing more sensitive and accurate diagnostics using a complementary and comprehensive set of metrics.

This Research Topic illustrates a new phase in research of ocular biomarkers of neurodegenerative diseases. Beyond showing retinal thinning in a patient group, investigation now focuses on deeper and more complex questions, such as: How do the retinal measures corroborate or complement existing clinical markers? How do they fit into the known disease models developed for the brain? Can we use the retina for testing and developing novel therapies? How do ocular functional changes correspond to retinal morphological changes in neurodegenerative or systemic diseases? We encourage future submissions on these and other Research Topics that will contribute to advancing our understanding and improving diagnosis and care of these devastating diseases.
